# Training Load Capacity, Cumulative Risk, and Bone Stress Injuries: A Narrative Review of a Holistic Approach

**DOI:** 10.3389/fspor.2021.665683

**Published:** 2021-05-28

**Authors:** Karrie L. Hamstra-Wright, Kellie C. Huxel Bliven, Christopher Napier

**Affiliations:** ^1^Department of Kinesiology and Nutrition, University of Illinois at Chicago, Chicago, IL, United States; ^2^Department of Interdisciplinary Health Sciences, Arizona School of Health Sciences, A.T. Still University, Mesa, AZ, United States; ^3^Menrva Research Group, Faculty of Applied Science, Schools of Mechatronic Systems Engineering and Engineering Science, Simon Fraser University, Vancouver, BC, Canada; ^4^Department of Physical Therapy, Faculty of Medicine, University of British Columbia, Vancouver, BC, Canada

**Keywords:** risk factors, relative energy deficiency in sport, patient-centered care, training load, assessment

## Abstract

Bone stress injuries (BSIs) are a common orthopedic injury with short-term, and potentially long-term, effects. Training load capacity, influenced by risk factors, plays a critical role in the occurrence of BSIs. Many factors determine how one's body responds to repetitive loads that have the potential to increase the risk of a BSI. As a scientific community, we have identified numerous isolated BSI risk factors. However, we have not adequately analyzed the integrative, holistic, and cumulative nature of the risk factors, which is essential to determine an individual's specific capacity. In this narrative review, we advocate for a personalized approach to monitor training load so that individuals can optimize their health and performance. We define “cumulative risk profile” as a subjective clinical determination of the number of risk factors with thoughtful consideration of their interaction and propose that athletes have their own cumulative risk profile that influences their capacity to withstand specific training loads. In our narrative review, we outline BSI risk factors, discuss the relationship between BSIs and training load, highlight the importance of individualizing training load, and emphasize the use of a holistic assessment as a training load guide.

## Introduction

Bone stress injuries (BSIs), specifically stress fractures, account for approximately 10% of all orthopedic injuries and as much as 20% of injuries managed in sports medicine clinics (Pegrum et al., 2014; Changstrom et al., 2015; Abbott et al., 2019a). As many as 22% are recurrent, require prolonged recovery (>3 weeks), and can often be season or career ending injuries (Wentz et al., 2011; Changstrom et al., 2015; Rizzone et al., 2017). These high rates and sequelae underscore the significant impact that BSIs have on both the healthcare system's ability to provide optimal care and individuals who experience a multitude of short- and long-term effects. In the short-term, BSIs cause pain, time loss from sport, and disability (Miller and Best, 2016; Abbott et al., 2019a). Long-term effects range from increased risk of re-injury, decreased bone health, and financial burden (Miller and Best, 2016; Rizzone et al., 2017; Abbott et al., 2019a). Therefore, it is incumbent on healthcare providers to reexamine current practices and strive for better outcomes in recognizing and managing athletes with BSIs.

While patients with BSIs represent a broad population, our focus is on athletes and military personnel; populations in which BSIs, particularly stress fractures, are common. Within sporting activities, the incidence of BSIs varies by sport, yet they most commonly develop during the regular season in sports requiring endurance and repetitive loading, such as running, gymnastics, and dancing (Rizzone et al., 2017). Collegiate athletes with the highest stress fracture injury rates are women's cross country, women's gymnastics, and women's outdoor track (Rizzone et al., 2017). The reported rates in college (5.70/100,000 athlete exposures) are higher than those in high school (1.54/100,000 athlete exposures), yet the pattern of high risk athletes is consistent (Changstrom et al., 2015; Rizzone et al., 2017). Military personnel, particularly recruits, during basic training are another unique population with a high prevalence of BSIs. There are two military recruit profiles at greatest risk for BSIs. Unfit, overweight, and sedentary recruits who are suddenly required to perform bouts of high intensity physical activity may quickly lose weight and nutrient stores, which puts them into an energy fatigue/insufficiency state so they are unable to repair and rebuild at the required rate (Wentz et al., 2011). The other profile consists of recruits more akin to athletes with good fitness levels, yet the abrupt changes in training load still exceed the body's recovery capacity (Wentz et al., 2011). Thus, an important consideration for healthcare providers and coaches to prevent BSIs and their reoccurrence is training load.

It is well-accepted that training load plays a critical role in the occurrence of BSIs. Clinically, the bone's response to loading occurs along a continuum from normal remodeling to accelerated remodeling, stress reaction, stress injury, stress fracture, and finally complete fracture (Bennell et al., 1999; Barrack et al., 2014; Kraus et al., 2019). A healthy bone's response to increased repetitive loads is to repair itself and re-establish its state of homeostasis, ultimately accelerating bone remodeling processes and strengthening itself against further insults (Bennell et al., 1999; Rizzone et al., 2017; Abbott et al., 2019a). However, if the bone's energy/nutrient stores are insufficient and the rest and recovery process is unable to keep pace with the repetitive loads, particularly at sites where stress is concentrated, the bone will fatigue, and BSIs develop and can progress in severity (Bennell et al., 1999; Miller and Best, 2016; Rizzone et al., 2017). As the bone is subjected to continued repetitive abnormal loads over a relatively short time, increased bone or marrow signaling signifies a stress reaction, which are followed by microcracks developing in the cortical layer of the bone (Bennell et al., 1999; Wentz et al., 2011; Rizzone et al., 2017; Kraus et al., 2019). As more and deeper microcracks develop, propagating along lines of stress, clinically symptomatic stress fractures manifest. If left unmanaged, these microcracks may progress into the most severe BSI, complete fractures (Bennell et al., 1999; Miller and Best, 2016; Rizzone et al., 2017; Abbott et al., 2019b). While clinical responses of the bone to loading are progressive along a continuum with specific diagnoses, practically, healthcare providers accept the encompassing term BSIs because one's approach to identifying risk factors and developing treatment programs is often the same regardless of the type of BSI.

Bertelsen et al. (2017) proposed a framework for the etiology of running-related overuse injuries that applies to the development of BSIs. In this framework, each tissue (i.e., bone) has a structure-specific load capacity, meaning that it can withstand a certain number of applied loads at a given magnitude over a given time before it fails. Overuse injuries are the result of the gradual reduction in the load capacity of certain structures following repeated stresses, with injury occurring when the structure-specific load capacity is exceeded by the cumulative training load applied to that structure. This framework highlights the delicate balance between load and capacity and the need to estimate, to the best of our abilities, the capacity of the structure by considering risk factors, as well as the applied training load, in order to prevent injury from occurring.

Many factors determine how an individual responds to the repeated stresses that have the potential to increase the risk of a BSI. As a scientific community, we have identified many isolated BSI risk factors occurring in athletes and military personnel (Bennell et al., 1996, 1999; Korpelainen et al., 2001; Lappe et al., 2001; Milner et al., 2006b; Kelsey et al., 2007; Pohl et al., 2008; Moran et al., 2013; Tenforde et al., 2013; Wright et al., 2015; Abbott et al., 2019a; Dixon et al., 2019; Nose-Ogura et al., 2019). However, risk factors do not exist in isolation. For example, individuals with higher training loads and lower bone mineral density (BMD) are at a greater risk of developing BSIs than those with higher training loads and normal BMD. Diet, hormone levels, stress, sleep, and other lifestyle factors may all interact and modify the risk of BSIs beyond a simple additive formula. As scientists, to date, we have not adequately analyzed the integrative, holistic, and cumulative nature of the risk factors. A combined approach is essential to determine an individual's specific load capacity. We have also not studied risk factors through the lens of training load. It is likely the integrative, holistic, and cumulative nature of risk factors vs. specific risk factors in isolation that explains why some individuals can train at a higher level than others without injury. It is also likely that an appreciation of the interactive nature of risk factors is essential to determine an individual's personal and specific training load capacity.

In this narrative review, we advocate for a personalized approach to training load so that individuals can optimize their health and performance. We propose that athletes and military personnel, referred to collectively as “athletes” going forward, carry their own cumulative risk profile that influences their training load capacity. We define a “cumulative risk profile” as a subjective clinical determination of the inherent risk factors with thoughtful consideration of how these risk factors interact with one another. This risk profile reflects the individual's capacity to tolerate training loads—or more specifically, to tolerate rapid changes in training loads. We outline BSI risk factors and discuss the relationship between BSIs and training load, how understanding risk factors helps to individualize training load, and the importance of a holistic assessment as a guide to individualize training load in athletes. We conclude with suggested screening tools for individuals who work with athletes to use during their holistic assessment to determine BSI risk.

## The Relationship Between BSI, Training Load, and Training Load Capacity

BSI is cumulative and multifactorial (Nattiv et al., 2007; Kraus et al., 2019). Training load and recovery are key factors in the development of BSIs since it is the loading of the bone without sufficient recovery time for remodeling that results in a BSI. The amount of training an athlete can withstand varies from one athlete to the next while also varying *within* an individual athlete over time (Nielsen et al., 2012). Training load in its most basic sense is a stress. Stress of all types, physical and non-physical, influence the human body. Physical stressors are often categorized as internal or external. Internal stressors are a measure of the athlete's response to external stressors and are quantified using variables, such as heart rate and rating of perceived exertion (RPE) (Jones et al., 2017; Paquette et al., 2020). Conversely, external stressors originate from outside of the body (e.g., application of mechanical loads), are characteristics of the environment the athlete participates in, and are often identified as extrinsic risk factors for BSIs (Bennell et al., 1999; Jones et al., 2017). Examples of extrinsic risk factors that impact how our body responds to external stressors in the form of distance covered or number of repetitions include footwear, training surface, or grade. While extrinsic risk factors are modifiable, intrinsic, or internal, risk factors are non-modifiable or modifiable, as they originate from within the individual and are characteristics of the athlete, such as age, sex, BMD, or hormone status (Bertelsen et al., 2017). Non-physical stressors are emotional or psychological in nature and occur as a result of lifestyle. These stressors can be moderated by daily routines, unexpected events, work hours, personality traits, family roles and responsibilities, and more. While it is clear that these non-physical stressors can affect one's emotional and psychological health, they also have a significant impact on physiological systems. The cumulative effect of these stressors can decrease an individual's load capacity, which may be reflected in their response to external loads (Paquette et al., 2020). It is, therefore, important to consider these factors and how they influence performance and health.

Consideration of both physical and non-physical stressors is important when determining the amount of training an athlete can withstand and benefit from, for purposes of training and performance, recovery, and injury management. Health and fitness professionals who understand how to modify training load and maximize recovery according to an athlete's individual physical and non-physical stressors, and then who empower athletes to make appropriate load and recovery decisions themselves, are critical in preventing injury and optimizing performance.

## Understanding Risk Factors to Individualize Training Load

### Intrinsic and Non-modifiable Risk Factors

Sex and age are intrinsic, non-modifiable risk factors for BSIs. The incidence of BSIs in female athletes is greater than that in male athletes (Wentz et al., 2011; Ruddick et al., 2019), but not uncommon for either sex (Changstrom et al., 2015; Rizzone et al., 2017). In a prospective study of female United States Army recruits, Black females were less likely to develop a BSI than White females during basic training (Lappe et al., 2001). In general, Black females and males have greater BMD and more favorable bone architecture (cortical and trabecular volumetric BMD and tibia microarchitecture) and strength even after adjusting for factors, such as age, weight, height, and physical activity (Popp et al., 2017). Similarly, as one ages, BSI risk may increase due to decreases in bone density and other individualized, multifactored reasons (Breer et al., 2012).

A largely unexplored but important area to research further and consider when working with athletes is the role of genetics in BSIs (Norwitz et al., 2019). How biological and environmental signals impact gene expression (epigenetics), and thus bone health, is also an area for further exploration that may elucidate the degree to which genetic factors may be more modifiable than what we currently understand based on existing evidence.

Another intrinsic, non-modifiable risk factor is skeletal alignment. Arch height, foot type, leg length, knee posture, and Q angle may play a role in putting an athlete at risk for BSIs (Sullivan et al., 1984; Simkin et al., 1989; Bennell et al., 1996, 1999; Miller and Best, 2016). In a small sample of 31 female and male athletes (19 long-distance runners and 12 from a variety of other sports), those who experienced multiple BSIs had a greater leg-length discrepancy, higher longitudinal arch, and greater forefoot varus than controls (Korpelainen et al., 2001). However, like previous studies on skeletal alignment as risk factor for BSIs, larger sample sizes are needed. The evidence to support skeletal alignment as a risk factor for BSIs remains inconclusive and contradictory.

Perhaps the strongest non-modifiable risk factor is prior bone injury. In a prospective study of female cross-country runners, those with a previous BSI had more than a five-fold higher rate of BSI, particularly stress fractures, during the average 1.85-year follow-up than females without such a history (Kelsey et al., 2007). Similarly, in a prospective study of female and male high school runners, females had a six-fold and males a seven-fold increased risk of the development of a stress fracture if they had a stress fracture history (Tenforde et al., 2013). Limited literature exists regarding why prior BSI puts individuals at risk for future injury; we propose that it is likely associated with an athlete's cumulative risk profile, which is discussed in-depth later in this paper.

### Intrinsic and Modifiable Risk Factors

Other intrinsic risk factors influencing training load and are modifiable include muscle strength and fatigue, flexibility, biomechanics, recovery strategies, nutrition, energy deficiency, and stress.

#### Muscle Strength and Fatigue

Soft tissue plays an important role in attenuating and absorbing force (Paul et al., 1978; Bennell et al., 1999), and thus muscle strength has the potential to reduce BSI risk (Hoffman et al., 1999). However, research investigating the direct relationship between muscle strength and BSI risk is limited, and the role of muscle fatigue on bone health is unclear. Hip abduction and external rotation strengthening are common injury prevention and rehabilitation focus when working with running athletes. The quality of research investigating hip strength on running-related injuries is low (Christopher et al., 2019), and the direct link between hip strength and BSIs has not been studied. Regarding muscle fatigue, in a biomechanical model analyzing strain on the tibia, fatigued calf musculature reduced bone strain, whereas fatigued thigh musculature increased bone strain (Hadid et al., 2018), revealing the complex relationship between muscle fatigue and bone strain. It is important to consider that muscle forces themselves account for a significant majority of the load applied to the bone (Scott and Winter, 1990). Therefore, the previous study's findings may be explained by a reduction in calf muscle forces that occurs as joint work shifts from distal to proximal with the onset of fatigue (Sanno et al., 2018). In the field, generalized training fatigue is a primary interest of clinicians and coaches alike. From a training perspective, running fatigue is a necessary side effect of improving performance. However, running fatigue alters biomechanics and may increase injury risk (Winter et al., 2017, 2020). An athlete whose physiological capacity is suboptimal relative to the training load being applied could be at risk of biomechanical changes that increase bone stress.

In summary, the relationship between muscle strength, muscle fatigue, and general training fatigue with BSIs needs more research. However, in the field, clinicians and coaches instinctively assess muscle strength, muscle fatigue, and training fatigue due to an understanding, grounded in experience, that these factors matter when it comes to BSI risk. Furthermore, high internal responses (e.g., RPE) to normal external loads may signify the need to increase rest and recovery for the athlete (Paquette et al., 2020). Until strong science directs otherwise, it makes intuitive sense to include muscle strength, muscle fatigue, and training fatigue as part of a holistic assessment when determining training loads and trying to prevent BSIs.

#### Flexibility and Range of Motion

Flexibility and resulting range of motion is determined by several factors, including ligamentous laxity, muscle length, and overall joint mobility (Bennell et al., 1999). Research examining flexibility, or lack of, related to BSI focuses on lower extremity joints, and results are inconsistent. Bennell et al. (1999) reviewed studies investigating the association between muscle and joint flexibility and BSIs and reported inconsistent findings regarding the association. For example, early work found an association between increased hip external rotation and BSIs in a population of Israeli infantry recruits (Giladi et al., 1987), whereas decreased ankle dorsiflexion motion was a risk factor in another study (Hughes, 1985). However, the association between increased hip external rotation, or ankle motion in any direction, with BSIs was not supported in more recent prospective studies on Royal Marines (Nunns et al., 2016; Dixon et al., 2019). Collectively, it is clear that the role of muscle and joint flexibility on BSI risk specifically in athletes is inconclusive and explanations are likely due to difficulties in standardization of terminology and assessment methods of flexibility and range of motion between studies. Given the plausibility of muscle and joint flexibility altering forces applied to the bone, and because it is a clinically practical measurement that can be monitored over time, it is a factor to consider when conducting a holistic clinical assessment.

#### Biomechanics

Abnormal biomechanics have been hypothesized to contribute to the development of BSIs (Miller and Best, 2016). Both abnormal kinetics and kinematics influence BSI occurrence (Warden et al., 2014) by subjecting the bone to loads it cannot withstand. Retrospectively, individuals with a history of BSI (tibia and femur) have demonstrated increased vertical ground reaction force loading rates compared with those without a history (Grimston et al., 1991; Milner et al., 2006a,b). Notably, loading rate is one factor related to a bone's fatigue limit (Milner et al., 2006b) and is possible to reduce via retraining (Napier et al., 2015).

Abnormal kinematics have also been associated with the development of BSIs. Female runners with a history of tibia BSIs displayed greater peak rearfoot eversion and hip adduction than female control runners (Milner et al., 2010). In military recruits while running in boots, peak rearfoot eversion occurred significantly earlier, and the angle of application of peak resultant horizontal force during braking was directed more laterally in those with a history of third metatarsal BSIs than in those without a history (Dixon et al., 2006). In a retrospective, predictive statistical analysis of female runners with a history of tibial BSIs, greater peak hip adduction, peak rearfoot eversion, and absolute free moment increased the likelihood of a participant having a previous BSI (Pohl et al., 2010).

Though research thus far points to increased vertical loading rate, hip adduction, and rearfoot eversion as key kinetic and kinematic risk factors for the development of BSI, it is important to note that the studies referenced in this section are retrospective in nature. Thus, it remains unknown whether these kinetic and kinematic variables should be addressed as an intervention in a clinical setting. To note, in a systematic review on running modification training programs, gait retraining interventions, such as foot strike manipulation and real-time feedback, may result in short-term biomechanical changes (Napier et al., 2015). However, it is unclear if these changes are beneficial for reducing injury and more research with longer term training and follow-up is needed (Winter et al., 2017). Foot strike manipulation, in particular, may actually increase the risk of running-related injury and therefore should be employed with caution (Anderson et al., 2020).

#### Recovery

One of the most current topics discussed among healthcare providers and athletes regarding its role in injury prevention, treatment, and performance is recovery (Kellmann et al., 2018). If an athlete can enhance recovery, their body will be more able to adapt to the loads inherent in sport, theoretically improving performance while keeping the athlete healthy (Kellmann et al., 2018). The emergence of wearable technologies and apps to track recovery metrics makes studying how recovery impacts healing and performance more feasible than before. In the field, athletes use a variety of recovery techniques, such as cryotherapy, thermotherapy, massage, and other forms of soft tissue work, such as foam rolling, mobility exercises, active recovery (light exercise), and more. In this paper, we focus on sleep and post-exercise nutrition as these are two of the more commonly discussed, accessible, and researched recovery strategies.

**Figure 1:**
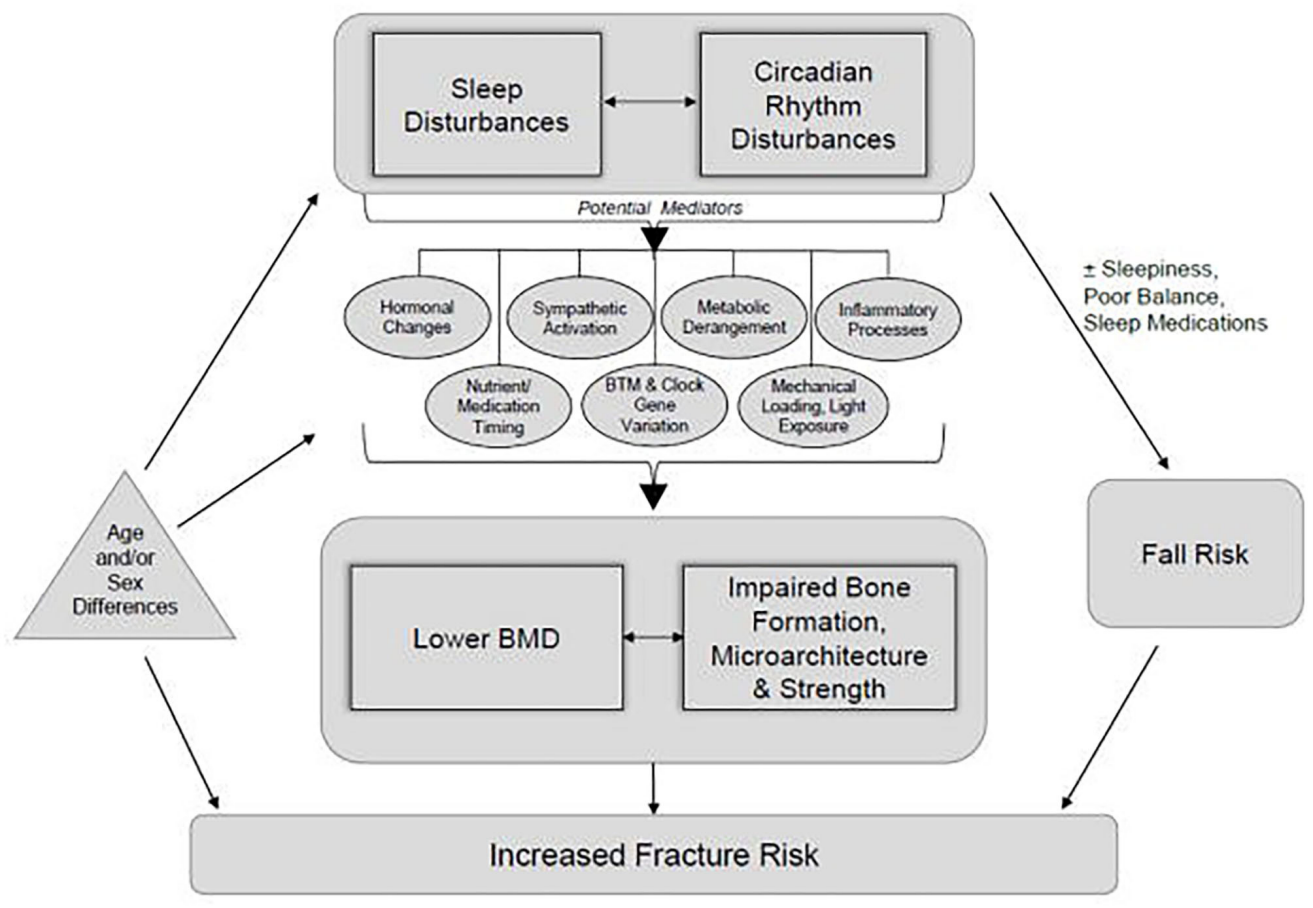
A conceptual framework demonstrating how disturbances in sleep and circadian rhythm have the potential to alter bone formation, microarchitecture, strength, and bone mineral density and thereby increase fracture risk. Although originally presented relative to populations outside of athletes, the framework offers insights regarding potential applications for bone stress injuries in athletes. Reprinted from Swanson et al. (2018), with permission.

Disturbances to sleep timing, sleep duration, or the biological processes normally served by sleep can interrupt the timing of bone turnover markers. This can disrupt the balance between bone resorption and formation, decreasing bone health and increasing fracture risk (Swanson et al., 2018). In a study of 95 endurance sport athletes who self-reported their sleep time over a 2-week period, those who slept <7 h/day were at a 51% increased risk of new injury, whereas athletes who slept >7 h/day reduced new injury risk by 37% (Johnston et al., 2020). Specific to BSI, in a population of 314 adolescent high school athletes who experienced 346 BSIs over a 2-year period, those with BSI reported sleeping less than those without a BSI (7.2 vs. 7.95 h/day) (Nussbaum et al., 2019). Figure 1, reprinted from Swanson et al. (2018) with permission, demonstrates the complex, yet important, intersection between sleep and bone health.

In addition to sleep, post-exercise nutrition is important in recovery and may be important for bone health as well. Post-exercise ingestion of carbohydrate and protein also aids recovery by restoring muscle glycogen (Kerksick et al., 2017; Kloby Nielsen et al., 2020). Post-exercise carbohydrate intake is especially important when rapid restoration of muscle glycogen is the goal and/or when an athlete's daily carbohydrate intake does not meet the energy demands of the sport (Kerksick et al., 2017). Of particular importance for endurance athletes is the impact that carbohydrates have on attenuating increases in inflammation and stress hormones (Nieman et al., 2003, 2005). The significance of attenuating exercise-related increases in stress hormones and the relationship between stress and the hypothalamus–pituitary–adrenal (HPA) axis are key considerations for endurance athletes and are further explained later in this paper. Specific to bone health, nutrient ingestion post-exercise can alter the response of bone turnover markers to that exercise session. For instance, in a small group of male runners who ran to exhaustion on a treadmill, immediate ingestion of a carbohydrate plus protein solution resulted in decreased bone resorption markers and increased bone formation markers and thus a more positive bone turnover balance (Townsend et al., 2017).

It appears that both sleep and post-exercise nutrition impact bone turnover markers. Bone turnover markers provide an indication of bone health, but do not measure actual BSI incidence. A needed next step is research evaluating if a direct relationship exists between sleep and BSIs as well as post-exercise nutrition and BSIs.

#### Nutrition and Energy Deficiency

In addition to post-exercise, other nutritional factors, specific to athletes, are important to consider as potential BSI risk factors, such as vitamin D, calcium, iron, ferritin, and energy deficiency. Vitamin D is a key modulator of bone remodeling (McCabe et al., 2012; Williams et al., 2020), and low levels appear related to BSI incidence as recently reported in other papers (Miller and Best, 2016; Abbott et al., 2019a). Although vitamin D's utility as a preventive measure, as well as what constitutes the optimal vitamin D level, is not clear, a recommended therapeutic goal for bone health is a serum 25-hydroxyvitamin D [25(OH)D] level of 50–100 nmol/L dependent on the individual (McCabe et al., 2012; Sale and Elliott-Sale, 2019). Recommendations specific to athletes are needed, but researchers studying iliac crest mineralization in a general population of 675 European adults (30–99 years) recommended 25(OH)D serum levels of above 75 nmol/L in conjunction with appropriate calcium intake bone health (Priemel et al., 2010).

Calcium is a key bone forming mineral and plays a major role in many cellular processes occurring during exercise (Sale and Elliott-Sale, 2019). Specific to athletes, during prolonged intense exercise, dermal calcium loss occurs, which could hypothetically decrease serum calcium levels, activate parathyroid hormone, and contribute to bone demineralization (Sale and Elliott-Sale, 2019). However, the amount of calcium lost in sweat would need to be significant to disrupt calcium homeostasis to the degree it would impact bone metabolism (Sale and Elliott-Sale, 2019). In contexts of significant sweat loss or dehydration, calcium supplementation could be beneficial. For instance, in male and female endurance cyclists, calcium supplementation just prior to a 90-min or 35-km trial attenuated an exercise-induced increase in markers of bone resorption (Barry et al., 2011; Haakonssen et al., 2015). Similarly, osteoclastic activity was suppressed after 60 min of cycling in male triathletes who supplemented with high calcium mineral water before and during the exercise bout (Guillemant et al., 2004). Intestinal calcium absorption is aided by vitamin D (Sale and Elliott-Sale, 2019), and thus supplementation of both may contribute to the prevention of BSIs. In a study of 3,700 female Naval recruits, those who supplemented with 200 mg of calcium and 800 IU of vitamin D had a 20% lower incidence of BSIs than the placebo group of female Naval recruits (Lappe et al., 2008). More research on various athlete groups is needed to further determine the preventive role of calcium and vitamin D supplementation on BSIs.

Iron supports vitamin D metabolism (Sale and Elliott-Sale, 2019) and, when deficient, appears to impact bone turnover markers. In a study of females 18–35 years, iron status was measured as ferritin, a biomarker of iron stores, and low iron status (serum ferritin <30 ng/ml, hemoglobin >11 g/dl) was associated with higher bone resorption (Toxqui et al., 2014). Iron deficiency is also related to BSI occurrence. In a group of female soldiers, at the end of basic training, those who incurred a BSI had higher rates of iron deficiency anemia and transferrin saturation deficiency than soldiers without a BSI (Yanovich et al., 2011). Iron deficiency influences multiple physiological systems and can be a contributing factor in relative energy deficiency in sport (RED-S) (Petkus et al., 2017).

RED-S is a syndrome describing impaired function of physiological processes including metabolic rate, menstruation, bone health, immunity, protein synthesis, cardiovascular health, psychological well-being, and more (Mountjoy et al., 2018). RED-S includes the female athlete triad, which is the interrelationship among low energy availability with or without disordered eating or an eating disorder, menstrual dysfunction, and low BMD (Nattiv et al., 2007; Mountjoy et al., 2018). Decreased energy availability impacts not only female athletes but also male athletes with more research on RED-S and male athletes needed (Tenforde et al., 2016; Mountjoy et al., 2018). In both females and males, low energy availability is likely to decrease sex hormones that then results in impaired bone health (Tenforde et al., 2016; Mountjoy et al., 2018). Estrogen is an especially critical hormone in maintaining bone mass in both sexes (Goolsby and Boniquit, 2017). For athletes with RED-S, the fundamental issue is a lack of adequate energy to support the range of body functions necessary for optimal health and performance (Mountjoy et al., 2018; Papageorgiou et al., 2018). As clinicians, identifying BSI risk relative to RED-S and the female athlete triad involves understanding the complex interaction of physiological and psychological contributors while simultaneously integrating the most current screening tools to gain insight from and about the athlete (De Souza et al., 2014). These screening tools are discussed later in this paper.

#### Bone Health

Specific to bone health, decreased energy availability and menstrual dysfunction are related to less BMD, altered bone microarchitecture, and changes in bone turnover markers in female athletes (De Souza et al., 2008; Ackerman et al., 2012; Papageorgiou et al., 2017, 2018). Although BMD is often used as a measure of bone health, bone strength and BSI risk depend not only on BMD but also on the internal structure of one's bone mineral, the quality of bone protein, and other bone health markers (Nattiv et al., 2007). Peripheral quantitative computed tomography (pQCT) can provide helpful measures of three-dimensional volumetric bone density, bone geometry, and muscle cross-sectional area. For instance, using pQCT to estimate strength, Popp et al. (2017) found that female distance runners with a history of BSI had significantly less tibia strength in relation to their mean active peak vertical ground reaction force during a fatiguing treadmill run than female distance runners without a history of BSI. Using similar methodology, male distance runners with a history of BSI have demonstrated smaller tibia and lower bone strength at the mid-diaphysis than male distance runners without a history of BSI (Popp, 2020). As bone health measures continue to evolve, more research on their relationship with BSIs will continue to guide practical and scientific applications (Nattiv et al., 2007).

#### Stress

Physical stress is needed during exercise for the body to adapt, grow, and perform. Psychological stress, within healthy limits, also has beneficial effects on personal growth. Interestingly, physical stress and psychological stress are perceived by the human body in similar ways. Both stressors activate two primary interrelated, yet distinct, systems: the sympathetic–adrenal–medullary (SAM) axis and the HPA axis (Clark and Mach, 2016; Godoy et al., 2018). Changes in physiological stress-related factors and psychosocial stress interact to increase the risk of injury, likely mediated by a reduction in an athlete's load capacity (Fisher et al., 2020). Positively, higher levels of physical activity and fitness may help attenuate psychosocial stress responses (Mücke et al., 2018). However, there may be a limit to which physical activity is beneficial (Luger et al., 1987; Hermann et al., 2019). For example, in a group of highly trained male runners, their evening basal plasma concentrations of adrenocorticotropic hormone and cortisol—measures that provide insight about the HPA axis—were elevated compared with untrained and moderately trained male runners (Luger et al., 1987). Short-term benefits of a hyperactive HPA axis could be detrimental when they persist chronically (which can occur without adequate recovery during training) due to immunosuppressive, catabolic, behavioral, and anti-reproductive effects (Luger et al., 1987).

Specific to BSIs, military recruits prospectively followed and diagnosed with stress fracture had a higher level of psychological stress than those who did not incur a stress fracture (Moran et al., 2013). In an animal model, stress activated the HPA axis, leading to increased serum corticosterone and ultimately bone loss by inhibiting osteoblasts and stimulating osteoclasts (Azuma et al., 2015b). While this psychological stress–bone mechanism has been largely studied using animal models and relative to osteoporosis in humans (Azuma et al., 2015a,b), dysregulation in bone metabolism due to chronic psychological stress is an important and novel risk factor to consider when assessing BSI risk.

In summary, the combined effect of physical and psychological stressors has the potential to increase injury more than any one factor in isolation (Fisher et al., 2020). Because the human body perceives physical and psychological stresses similarly, it is important to assess the impact of both physical stress and psychological stress on athletes, its possible contributions to injury, and the critical role of recovery from the compounded impact of both types of stressors (Nattiv et al., 2007; Wiese-Bjornstal, 2010; Putukian, 2016). The positive stress of exercise could become harmful if the body remains in an ongoing state of stress due to insufficient or inappropriate recovery. Theoretically, physical and psychological recovery would allow the body to resume its resting state before the next bout of stress and aid the athlete's health and performance. Suggested screening tools for stress and recovery are provided later in this paper that allow for the important integration of the multifaceted sources of stressors athletes encounter (Fisher et al., 2020).

### Intrinsic and Unknown Risk Factors

#### Gut Health

One emerging intrinsic risk factor, with unknown modifiability, is the relationship between the gut and bone health. The gut microbiota has received a lot of attention in recent years. The research on the inter-relationship between psychological stress, exercise, and the gut microbiota is limited but growing (Clark and Mach, 2016). A common thread shared between psychological stress, exercise, and the gut microbiota is the HPA axis. Gut microbial composition is important for the development and function of an appropriate HPA stress response (Sudo et al., 2004; Clark and Mach, 2016). The gut microbiome also influences bone formation (Ibáñez et al., 2019). When the gut microbiota is imbalanced, it is called dysbiosis, and gut dysbiosis has pathophysiological consequences (Baker et al., 2017). Specifically, with gut microbiome changes, greater dissemination of bacteria-derived compounds occurs along with alterations in the expression of cytokines and growth factors (Ibáñez et al., 2019). These alterations are believed to impair immune, endocrine, vascular, and nervous system responses that all play a role in regulating bone cell differentiation and/or function (Ibáñez et al., 2019).

For athletes who already have decreased levels of sex hormones (Tenforde et al., 2016) and who challenge the integrity of the gut epithelial barrier due to the nature of sport (Ticinesi et al., 2019), the relationship between sex hormones and gut health is particularly important to consider. In animal studies, sex hormones have been found to play an important role in the integrity of the gut epithelial barrier (Homma et al., 2005). Reduced gut microbiota diversity contributes to a decrease in estrogen metabolism because of a lack of estrogen metabolizing bacteria and other metabolic effects (Baker et al., 2017). Without estrogen metabolizing bacteria, a decrease in circulating estrogens results (Baker et al., 2017). As described earlier in our paper, decreased estrogen has detrimental effects for athletes. It is difficult to know with athletes which comes first, poor gut health leading to low sex hormones or low sex hormones leading to poor gut health. In the end, both situations could put an athlete at risk for a BSI due to the potential of gut dysbiosis impacting bone formation and sex hormone levels or low sex hormones impacting the gut and thus altering bone formation negatively. As research on the gut microbiome specific to athletes continues to develop, it will be interesting and important to study the relationship between gut and bone health.

#### The HPA Axis

A brief review of the intrinsic risk factors outlined in this paper highlights the role of the HPA axis in several of the risk factors: RED-S, stress, the gut microbiome, and bone health. Specific to RED-S, in female athletes with low energy availability, the hypothalamic–pituitary–gonadal (HPG) axis is also disrupted (Mountjoy et al., 2018). Similarly, male endurance athletes often have low testosterone levels that may be linked to alterations in the HPG axis (Tenforde et al., 2016). The HPG and HPA axes are strongly connected and critical for reproduction and survival (Acevedo-Rodriguez et al., 2018). As described in the preceding paragraphs, chronic psychological and physical stress disrupts the HPA axis, and gut microbial composition appears crucial for a healthy HPA stress response. Increased HPA axis activation has the potential to disrupt bone remodeling (Azuma et al., 2015b) and thus is an important consideration regarding BSI prevention and rehabilitation. Additionally, prolonged energy deficiency, as observed in RED-S, is thought to reduce intestinal function via mucosal atrophy, linking RED-S to gastrointestinal issues (Shaw et al., 2012; Melin et al., 2014). The interconnectedness of RED-S, stress, gut health, exercise, and BSIs is an important, emerging, and novel area to be explored by clinicians and researchers working together. Having a more scientific understanding regarding the relationship among these variables would give clinicians the opportunity to treat athletes with a greater holistic understanding of the multi-layered factors involved with bone health in athletes.

### Extrinsic and Modifiable Risk Factors

Notably, training loads can be modified not only by intrinsic factors but also by extrinsic factors, such as footwear, surface, number of strides/cycles, magnitude of load (kinetics), and distribution of load (kinematics) (Bertelsen et al., 2017; Paquette et al., 2020). Various extrinsic and modifiable risk factors are often discussed when treating BSIs in runners; however, few are supported in the literature (Wright et al., 2015). Theoretically, footwear (shoes, orthotics) can attenuate force and/or influence kinematics, but the ability of footwear to decrease BSI risk is unclear (Warden et al., 2014). While some have claimed that increased cushioning can protect from increased bone loads, others have claimed that minimalist shoes are the answer by reducing vertical loading rates (Rixe et al., 2012; Warne and Gruber, 2017). However, there is currently little evidence to support that any type of footwear can prevent running-related injuries, let alone BSIs (Napier and Willy, 2018). Similarly, it is thought that a harder training surface increases loading relative to a softer one and thus may be a risk factor for BSIs, but the training surface and BSI risk relationship remains unclear and complex (Warden et al., 2014). For instance, one must also consider variables, such as leg stiffness, running distance, and surface accommodation, among many other variables, when determining the role of surface type on BSIs (Warden et al., 2014). Runners can modulate leg stiffness depending on running surface, which may mitigate the effect of hard surfaces or cushioned shoes (Ferris et al., 1998, 1999).

Perhaps the most important risk factor to consider is training load. Generally speaking, overuse injuries occur when running intensity and/or volume increases at a pace the body cannot accommodate (Hreljac, 2005; Bertelsen et al., 2017). When considering training load as a risk factor for BSIs, factors to further research and consider clinically include the acute change in training load, cumulative training load, monotony strain, and acute:chronic workload ratio (Jones et al., 2017). While it is unclear as to exactly how much is too much, rapid increases in training loads have been associated with increased risk of injury, including BSI (Rauh, 2014; Damsted et al., 2018). Training load recommendations should be individualized as individual capacity to withstand changes in training load varies considerably (Nielsen et al., 2012). Demands of the sport should also be taken into account. For instance, high BSI-incidence sports, such as running and gymnastics, may be more prone to sudden changes in training load due to the greater bone loading in these sports combined with population-based characteristics, such as menstrual dysfunction and RED-S (Robinson et al., 1995; Rizzone et al., 2017). Conversely, swimming and cycling do not place significant loads on the bones and, therefore, may not be as prone to rapid changes in training load (Rizzone et al., 2017). A further important consideration is the method used to quantify training load. Recent studies have demonstrated that changes in training load can be substantially underestimated from week to week when not incorporating a measure of internal load (Napier et al., 2020; Ryan et al., 2020). Furthermore, the addition of a more biomechanically specific measure of load (e.g., cumulative shock) may have benefit when estimating the mechanical load applied to the body (Napier et al., 2020). Wearable technology is currently available to provide these metrics. However, it is not possible for any currently available wearable sensors to accurately capture bone-specific loads, and the accuracy of many of these devices is either unknown or questionable (Willy, 2018; Moore and Willy, 2019).

The reason why some individuals can accommodate a higher workload than others is multifactorial, but we propose that it is related to each athlete's cumulative risk profile, or load capacity. When possible, healthcare providers and coaches working together have the potential to individualize training load for athletes. They also have the opportunity to educate athletes on how to adjust their own training load or recovery strategies based on their unique cumulative risk profile to prevent BSIs and optimize performance. Developing a cumulative risk profile requires a holistic assessment with a keen understanding and appreciation of how the various risk factors interact.

## Conducting a Holistic Assessment as a Guide for Individualizing Training Load

A holistic assessment is essential to determine the optimal training load for each individual and also helpful to conclude when a referral to a specialist may benefit the athlete. Most studies have examined BSI risk factors independently instead of assessing their interdependence and relatedness (Wright et al., 2015), but it is critical to think of risk factors for BSIs as interacting vs. occurring in isolation when determining an athlete's capacity. For example, in male and female collegiate runners, it is the combined risk factor profile of low energy availability, low body mass index, prior BSI, and low BMD that most puts males at risk for a BSI (Kraus et al., 2019) with the addition of menstrual dysfunction that puts females most at risk (Tenforde et al., 2013).

As noted earlier in the paper, we define a “cumulative risk profile” as a subjective clinical determination of the number of factors putting an athlete at risk along with thoughtful consideration regarding how all the risk factors interact with one another. Making this clinical determination requires an evidence-based approach where the healthcare provider integrates the current literature, their clinical experience, and the athlete's state and preferences (Sackett et al., 1996). Ideally, as research develops and we learn more about the various BSI risk factors, precision medicine interventions (Jameson and Longo, 2015) and/or development of an objective cumulative risk score or risk assessment model will help in determining each individual athlete's optimal training load. Risk scores and risk assessment models have been created for the female athlete triad (Joy et al., 2014) and RED-S (Mountjoy et al., 2015) and could guide this approach for BSIs. Developing such tools, as well as precision medicine interventions, requires the collaborative efforts of clinicians and scientists simultaneously evaluating the holistic, integrated, and complex nature of BSI risk.

Specifically, we need more clinicians to subjectively assess cumulative risk profiles in athletes, and we concurrently need more scientists to evaluate the interdependence of risk factors. One of the challenges of determining which athletes are truly most at risk is conducting prospective studies that evaluate risk factor interdependence (Wright et al., 2015). Barrack et al. (2014, 2017) have begun important prospective work supporting the cumulative nature of risk factors for BSIs and BMD in females and males. However, more research is needed to support the work by Barrack et al. and on various populations (adolescents through master's athletes) with the understanding that as training years increase, so does one's individual cumulative risk. Having a better insight into the cumulative risk profile of athletes, along with stronger research examining risk factor interdependence, would provide greater understanding regarding why some athletes can withstand more training load than other athletes and thus allow for individualized training loads, optimized recovery, and enhanced performance.

**Figure 2:**
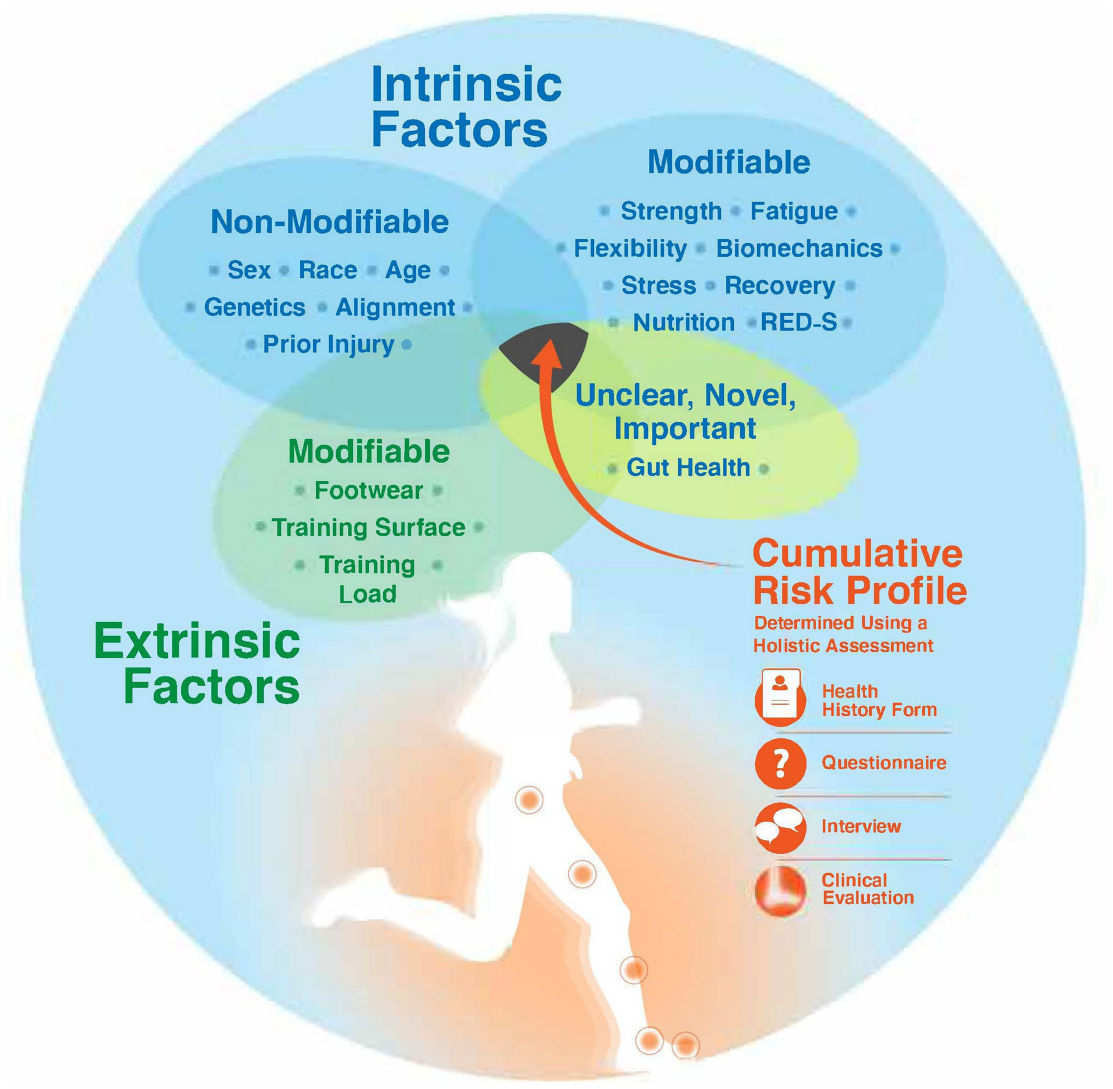
Determining an athlete's cumulative risk profile requires a holistic assessment of key intrinsic risk factors (modifiable and non-modifiable), extrinsic risk factors, and a risk factor with unknown modifiability (gut health). A holistic assessment includes utilization of a detailed health history form, questionnaires, a patient-centered and empathetic interview, and a clinical evaluation. RED-S, relative energy deficiency in sport.

Until a cumulative risk score or risk assessment model exists, determining an athlete's cumulative risk profile requires a holistic assessment based on the key intrinsic and extrinsic risk factors presented in this paper (Figure 2). The holistic assessment opens up the opportunity to individualize training load based on the athlete's unique risk. Practically, a holistic assessment can take place anytime when working with an athlete; however, pre-participation exams, clinical evaluations, or intake forms with an athlete in a performance-based setting represent opportune times. A thorough health history form, with questionnaires and an accompanying interview, and a clinical evaluation (if within the professional's standard of care) are important components of a holistic assessment.

Some key considerations for a health history form include basic questions on sex, ethnicity, age, genetics (family history), and prior bone injury. Additionally, inquiries about sleep duration and quality, post-exercise nutrition (timing and consumption), hydration, gut dysbiosis, and detailed questions about running history and current training loads (frequency, intensity, time, type) are essential components of a thorough health history form. Until an athlete-specific questionnaire on gut health exists, gut dysbiosis signs and symptoms to inquire about include nausea, vomiting, abdominal angina, (bloody) diarrhea, and more (de Oliveira et al., 2014).

Questionnaires to consider as part of the holistic assessment include: Athlete Sleep Behavior Questionnaire (Driller et al., 2018), Low Energy Availability in Females Questionnaire (LEAF-Q) (Melin et al., 2014), Relative Energy Deficiency in Sport Clinical Assessment Tool (RED-S CAT) (Mountjoy et al., 2015), Triad Cumulative Risk Assessment Tool (De Souza et al., 2014; Koltun et al., 2020), and the Recovery-Stress Questionnaire for Athletes (Kellmann and Kallus, 2001). These questionnaires provide windows into the athlete's recovery, energy availability, and stress levels. Notably, the Triad Cumulative Risk Assessment Tool can be adapted to use when assessing males (Heikura et al., 2018).

Many options exist regarding what to include in a clinical assessment. Minimally, examining levels of vitamin D, calcium, and ferritin as well as evaluating leg length discrepancy, muscle strength and endurance, current training status, and conducting a biomechanical analysis can provide professionals with a strong pulse on the athlete's physical presentation that they can then integrate with their health history, questionnaire, and interview learnings. If accessible, more advanced measures of nutrient status, hormone levels, bone health (turnover markers, density, strength, geometry, microarchitecture), and muscle cross-sectional area will greatly inform the holistic picture of the athlete's health.

BSIs typically are not strictly musculoskeletal injuries and involve a bodily systems approach to treat the root issue. Having a network of clinicians to refer athletes to or consult with in other areas, such as endocrinology, gastroenterology, nutrition, rehabilitation, performance, physiology, psychology, and more, can be a critical piece in restoring holistic health to the athlete. For instance, a focus on an athlete's psychological approach to their well-being and overall mental health can be especially important in cases presenting with RED-S (Mountjoy et al., 2018) and in cases where an athlete's psychological response to injury triggers mental health concerns, such as anxiety, depression, eating disorders, or substance abuse (Putukian, 2016). As research and clinical care continues to better understand the complex interactions between the bodily systems as it relates to bone health, having a network of professionals focused on the athlete will be important in determining each athlete's unique cumulative risk profile.

As clinicians adopt a holistic and patient-centered assessment approach, it is also important to consider strategies to improve the patient–clinician relationship to promote the best treatment outcomes. Empathy is a critical component of patient-centered care and one of the most important determinants in patient satisfaction (Hush et al., 2011; David and Larson, 2018). In a study of Division 1 student-athletes, student-athletes reported advocacy, approachability, and communication as important expressions of clinician empathy (David and Larson, 2018). Conducting a holistic assessment using an empathetic approach ensures patients feel that they are represented, listened to, and empowered, which is critical to developing an integrated and personalized training plan (David and Larson, 2018).

## Conclusion

In this narrative review, we outlined BSI risk factors—intrinsic and extrinsic, modifiable and non-modifiable—with an exploration of the new and emerging risk factor area of gut health. We discussed the relationship between BSIs and training load, emphasized how understanding risk factors helps individualize training load capacity, and highlighted the importance of a holistic assessment as a guide for individualizing training load. We also introduced the term “cumulative risk profile” as it relates to BSI prevention and optimization of training load and have provided recommendations of screening tools for clinicians working with athletes to use during their holistic assessment. It is clear that BSIs occur as a result of the interaction and interrelationship between multiple risk factors. As clinicians, relying on a thorough, holistic assessment is an important first step to prevent BSIs. After an assessment, a team-based approach in which all the health and fitness professionals working with an athlete are aware of the athlete's cumulative risk profile can help optimize the athlete's training load, recovery, and performance. Due to the many athletes that clinicians oversee, of particular importance is educating athletes so they are aware of their body's cues and have the confidence to use their voice to influence training load and recovery decisions. From a research perspective, we need prospective studies of female and male athletes that investigate the interrelationships between risk factors so that we have a clearer understanding of how multiple risk factors interact and influence an athlete's cumulative risk profile. Advances in precision medicine and risk assessment modeling will significantly assist scientific efforts in building an individualized cumulative risk profile for athletes.

## Author Contributions

KH-W and KHB contributed to the conception and design of the study, and wrote the first draft of the manuscript. CN significantly edited the subsequent drafts. All authors read and approved the submitted version.

## Conflict of Interest

The authors declare that the research was conducted in the absence of any commercial or financial relationships that could be construed as a potential conflict of interest.
